# Supplementary material for: Molecular Characterization and Cross-Reactivity of Feline Calicivirus Circulating in Southwestern China
Source: Viruses. 2021 Sep 12;13(9):1812. doi: 10.3390/v13091812 (PMC8473038; doi:10.3390/v13091812)
Supplement: Supplementary file 1 [file viruses-13-01812-s001.zip › viruses-1367179-supplementary.pdf]

Table S1. Primers used to amplify the VP1 HV and full-length genome of FCV strains in this study.

Primer name[a.]	Primers sequence (5´-3´)	Location[b]	Length (bp)
FCV-VP1-F	TTTATGTTGCTTGGTCTGG	5933-7262	1329
FCV-VP1-R	ATTCCCATGTAGGAGGCA		
FCV-A-F	GTAAAAGAAATCTGAGACAATG	1-2789	2788
FCV-A-R	AAGATTTCAGACCATGGC		
FCV-B-F	GGTGCTCGTATCTGGGC	2665-4797	2132
FCV-B-R	GTAGACACCATCATCACCAT		
FCV-C-F	TGTCTGTATGTTGGGTG	4683-5961	1278
FCV-C-R	CTCCACAGACCCAGACC		
FCV-D-F	AACCTGCGCTAACGTGCTTA	5350-6274	944
FCV-D-R	CAGTGACAATACACCCAGAAG		
FCV-E-F	TTTATGTTGCTTGGTCTGG	5933-7262	1329
FCV-E-R	ATTCCCATGTAGGAGGCA		
FCV-F-F	GGCATGTACGTCTGTGGG	6706-7683	977
FCV-F-R	CACTGGGGTTAGACGC		

[a] F and R represent forward and reverse primers, respectively. [b] The location is according to the genomic sequences of FCV-21223 strain (GenBank no. JX519212).
